# Ultrastructural changes in the retinopathy, globe enlarged (*rge*) chick cornea^☆^

**DOI:** 10.1016/j.jsb.2009.01.009

**Published:** 2009-05

**Authors:** Craig Boote, Sally Hayes, Robert D. Young, Christina S. Kamma-Lorger, Paul M. Hocking, Ahmed Elsheikh, Chris F. Inglehearn, Manir Ali, Keith M. Meek

**Affiliations:** a Structural Biophysics Group, School of Optometry and Vision Sciences, Cardiff University, Maindy Road, Cardiff CF24 4LU, UK; b The Roslin Institute and Royal (Dick) School of Veterinary Studies, University of Edinburgh, Roslin BioCentre, Midlothian EH25 9PS, UK; c Ocular Biomechanics Group, Division of Civil Engineering, University of Dundee, Dundee DD1 4HN, UK; d Leeds Institute of Molecular Medicine, University of Leeds, St. James’s Hospital, Leeds LS9 7TF, UK

**Keywords:** Corneal stroma, Chick, Collagen fibril, Biomechanics, Proteoglycan

## Abstract

In the cornea, the precise organisation of fibrillar collagen and associated proteoglycans comprising the stromal extracellular matrix plays a major role in governing tissue form and function. Recently, abnormal collagen alignment was noted in the misshapen corneas of mature chickens affected by the retinopathy, globe enlarged (*rge*) mutation. Here we further characterize corneal ultrastructural changes as the *rge* eye develops post-hatch. Wide-angle X-ray scattering disclosed alteration to dominant collagen lamellae directions in the *rge* chick cornea, compared to age-matched controls. These changes accompanied eye globe enlargement and corneal flattening in affected birds, manifesting as a progressive loss of circumferential collagen alignment in the peripheral cornea and limbus in birds older than 1 month. Collagen intermolecular separation was unchanged in *rge*. However, small-angle X-ray scattering results suggest collagen fibril separation and diameter increase more rapidly towards the corneal periphery in *rge* at 3 months post-hatch compared to controls, although central collagen fibril diameter was unchanged. By transmission electron microscopy utilising cuprolinic blue stain, the morphology and distribution of stromal proteoglycans were unaltered in *rge* corneas otherwise demonstrating abnormal collagen fibril organisation. From a numerical simulation of tissue mechanics, progressive remodelling of stromal collagen in *rge* during globe enlargement post-hatch appears to be related to the corneal morphometric changes presented by the disease.

## 1 Introduction

**Fig. 1:**
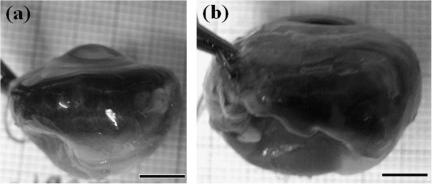
Globe enlargement and corneal flattening in *rge*. (a) Normally sighted control eye at 9 months post-hatch. (b) Age-matched *rge* eye. Scale bars: 5 mm.

Animal vision diseases in which normal eye growth is compromised can provide useful opportunities for determining the factors that govern the size and shape of ocular components. Retinopathy, globe enlarged (*rge*) is a recessively inherited condition of chickens (Curtis et al., 1987, 1988), arising from an in-frame 3 bp deletion in the cone β-transducin gene *GNB3* (Tummala et al., 2006) and resulting in protein destabilisation and primary retinal lesion. *Rge* is characterized clinically by progressive retinal degeneration and total visual loss by ∼1 month post-hatch (Inglehearn et al., 2003; Montiani-Ferreira et al., 2003, 2005). Morphometrically, the *rge* phenotype is defined by eye globe enlargement and associated thickening and flattening of the cornea occurring secondary to retinal dysfunction (Montiani-Ferreira et al., 2003; Inglehearn et al., 2003) (Fig. 1). A significant increase in radial globe diameter is apparent approximately 1 month post-hatch, with axial eye elongation following around 2 months later (Montiani-Ferreira et al., 2003). Corneal radius of curvature is also significantly increased by 1 month, but anterior chamber depth becomes notably shallower before this (Montiani-Ferreira et al., 2003). Recently, changes to collagen fibril organisation were observed in the corneal stroma of 9 month old chickens homozygous for *rge* (Boote et al., 2008).

In the cornea, as in other connective tissues, the arrangement of collagen in the extracellular matrix is a major determinant of tissue biomechanics (Hukins, 1984; Jeronimidis and Vincent, 1984; Hukins and Aspden, 1985; Meek, 2008). The bulk of mechanical loading on the cornea is borne by type I fibrillar collagen in the stroma, arranged in a layered structure that is unique amongst connective tissues (Maurice, 1957; Komai and Ushiki, 1991). In the mature chicken, these stromal layers, or lamellae, are 0.25–1.5 μm thick and are comprised of parallel, uniformly thin fibrils which lie within the plane of the tissue (Lucio and Smith, 1984). Specific interactions between collagen and proteoglycans located in the interfibrillar space are thought to be essential in regulating corneal fibril separation and diameter (Quantock and Young, 2008). In the central cornea, collagen lamellae are disposed in a fan-like pattern throughout the most anterior 1/4 of the stromal depth, but form a predominantly orthogonal network in the deeper layers (Lucio and Smith, 1984). Moreover the disposition of lamellar directions varies with position across the cornea: while depth-averaged fibril orientation is predominantly orthogonal in the central cornea, circumferentially-aligned collagen dominates in the periphery (Boote et al., 2008).

It is suspected that the highly specific architecture of the stroma is important in the maintenance of corneal shape and, since the cornea performs the bulk of light focussing in terrestrial vertebrates, in determining visual quality. Alterations to both stromal collagen alignment (Daxer and Fratzl, 1997; Quantock et al., 2003; Meek et al., 2005; Hayes et al., 2007) and proteoglycans (Funderburgh et al., 1989; Pellegata et al., 2000; Liu et al. 2003) have been widely implicated in conditions where normal corneal curvature and/or thickness are compromised. However, exactly how the precise corneal contour is achieved and maintained under the action of the intraocular pressure and external mechanical stresses remains poorly understood. Previously we identified changes in preferential collagen fibril orientation in the corneas of *rge* chickens at 9 months post-hatch, which we have hypothesized may relate to the corneal flattening observed in these birds (Boote et al., 2008). In the current study, we more comprehensively define ultrastructural changes in the *rge* cornea at earlier post-hatch time-points, using X-ray scattering methods to map collagen fibril orientation, spacing and diameter across the cornea. In addition, we utilise transmission electron microscopy with cuprolinic blue staining to visualise stromal proteoglycans. The observed stromal restructuring is considered in relation to tissue stress alterations simulated numerically from the accompanying reshaping of the *rge* eyeball.

## 2 Materials and methods

### 2.1 Animal details

The derivation and maintenance of the *rge* line at Roslin has been described previously (Inglehearn et al., 2003). The *rge* gene was backcrossed into a line of White Leghorn chickens that are also maintained at the Roslin Institute (Edinburgh, UK) and are used as the sighted controls. All husbandry and experimental techniques are performed under a Home Office project licence in accordance with the ARVO statement for the Use of Animals in Ophthalmic and Vision Research. Birds are floor-reared with a daily photoperiod of 14 h light and 10 h darkness, and transferred to individual cages at 16 weeks of age for subsequent pedigree breeding.

### 2.2 Tissue harvesting

Homozygous (*rge/rge*) blind White Leghorn chickens and age-matched, normally-sighted birds were euthanized with an overdose of sodium pentabarbitone at 1 and 3 months post-hatch. Immediately after death the eyes were enucleated and scleral sutures added to mark the superior globe position. Two normal and two *rge* eyes from the 3 month age group were taken and the corneas excised such that 2–3 mm of surrounding scleral tissue was retained. The excised corneas were initially transferred to 2.5% glutaraldehyde fixative (in 25 mM sodium acetate/0.1 M magnesium chloride buffer, pH 5.7), containing 0.05% cuprolinic blue, in order to stabilise the corneal curvature and minimise lamellar crimping upon subsequent dissection. After 30 min 1 × 2 mm full-thickness specimens were obtained from the central and peripheral cornea, returned to stain/fixative and stored at 4 ^o^C for transmission electron microscopy. All remaining eyes were snap frozen immediately after death with liquid nitrogen and stored at −80 ^o^C for X-ray experiments. Immediately prior to X-ray exposure these eyes were thawed and the corneas excised, again retaining a 2–3 mm rim of surrounding sclera. It has been previously shown that freezing and thawing corneas does not affect structural parameters as measured by X-ray diffraction (Fullwood and Meek, 1994).

### 2.3 Wide-angle X-ray scattering (WAXS)

**Fig. 2:**
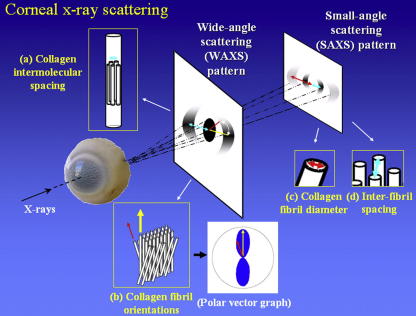
Structural information obtainable by wide- and small-angle X-ray scattering. (a) The radial position of the WAXS reflection can be used to determine average collagen intermolecular spacing. (b) The WAXS azimuthal intensity profile yields information on collagen orientation. The length of a particular vector in the resulting polar graph is indicative of the number of fibrils preferentially aligned in the vector direction. The radial position of SAXS reflections indicates the average collagen fibril (c) diameter, and (d) spacing.

WAXS experiments were performed on station 14.1 at the UK Synchrotron Radiation Source (Daresbury, UK). WAXS patterns were recorded at 0.4 mm intervals across *rge* and control corneas. Data collection followed the protocol described by Boote et al. (2008), using an X-ray beam of wavelength 0.1488 nm and 0.2 mm × 0.2 mm square cross-section at the specimen. Sample-to-detector distance was 15 cm and X-ray exposure time per data point was 20–30 s. For each WAXS pattern the normalized azimuthal intensity distribution around the collagen intermolecular reflection was measured as described previously (Aghamohammadzadeh et al., 2004), in order to obtain polar plots indicating the orientation of preferentially aligned collagen at each sampled point in the tissue (Fig. 2). In addition, WAXS patterns at 1 mm intervals along the medial-lateral corneal meridian were used for analysis of collagen molecular separation. The radial position of the intermolecular X-ray peak was calibrated against the 0.304 nm reflection of powdered calcite in order to calculate collagen intermolecular Bragg spacing (Fig. 2). Statistical analysis was performed using two-tailed *t*-tests.

### 2.4 Small-angle X-ray scattering (SAXS)

As shown in Fig. 2, SAXS can be used to determine corneal structural parameters including the average separation and diameter of collagen fibrils (Meek and Quantock, 2001). Corneal SAXS patterns were collected on station 2.1 at the UK Synchrotron Radiation Source (Daresbury, UK). Excised corneas were wrapped in cling-film in order to limit tissue dehydration and mounted inside airtight Perspex (Databank, UK) cells. X-rays (wavelength 0.154 nm) were passed through the anterior face of the cornea parallel to the optical axis and the resulting SAXS data collected on a detector placed 7.5 m behind the specimen. The X-ray beam was focussed to produce a square cross-section at the specimen measuring 1 mm × 1 mm. SAXS patterns were recorded at 1 mm intervals along the medial-lateral corneal meridian, the specimens being translated between exposures by means of a motorized stage interfaced with the X-ray camera shutter. The X-ray exposure time per data point was 90 s. The data analysis protocol has been described in detail previously (Boote et al., 2003a). In brief, collagen interfibrillar Bragg spacing was computed from the radial position of the corneal interference function peak, with calibration being achieved using the 67 nm meridional reflection from hydrated rat tail tendon. Bragg spacing was converted to centre-to-centre fibril spacing via multiplication by a packing factor of 1.12, which assumes the arrangement of stromal fibrils approximates the short-range order of liquids (Worthington and Inouye, 1985). Collagen fibril diameter was determined from the position of the fibril transform peak, assuming cylindrical collagen fibrils (Oster and Riley, 1952; Worthington and Inouye, 1985), and calibrated using hydrated rat tail tendon. Statistical significance was tested using two-tailed *t*-tests.

### 2.5 Transmission electron microscopy (TEM)

For visualization of proteoglycans, central and peripheral corneal specimens were allowed to incubate for several days at 4 ^o^C in glutaraldehyde fixative containing 0.05% cuprolinic blue (Scott et al., 1981). Contrast enhancement of stained proteoglycan–dye complexes was achieved by 15 min washes in, firstly aqueous, and then 1:1 aqueous/ethanolic, solutions containing 0.5% sodium tungstate. Specimens were then dehydrated in an ascending series of ethanol concentrations and subjected to 30 min propylene oxide and 60 min 1:1 propylene oxide/Araldite resin incubations, prior to resin embedding. Ultrathin sections (∼100 nm thick) were cut using a diamond knife, collected on copper grids and stained with uranyl acetate, before examination in an EM208 transmission electron microscope (Philips, Eindhoven, The Netherlands).

### 2.6 Numerical prediction of eye stress distribution

**Fig. 3:**
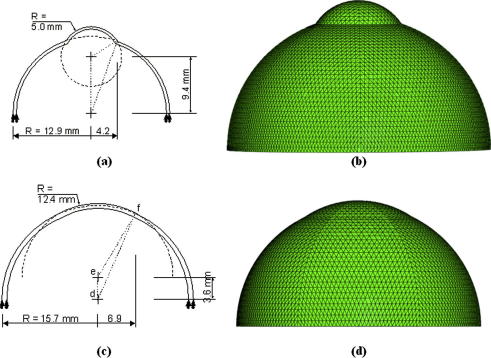
Geometric details of numerical simulations of (a,b) wild-type and (c,d) *rge* chicken eyes including the dimensions (a,c) and finite element meshes (b,d) used.

Numerical simulations, based on nonlinear finite element analysis, were carried out to relate the eyeball reshaping presented by *rge* to the stromal restructuring observed in the current study. Two simulations of wild-type and *rge* chicken eyes at 9 months were created, while considering dimensions estimated from digital images of whole excised eyes prior to dissection using PaintShop Pro 10.03 software (Corel, UK), as shown in Fig. 3a and c. The simulations considered only the anterior half of the eyeball and assumed that the sclera was allowed to deform radially, but not longitudinally, along the equator. Intraocular pressure (IOP) of 15 mm Hg was applied on the models’ inside surface, while monitoring the resulting stress distribution. The simulations employed 498 six-noded solid elements arranged in one layer, as shown in Fig. 3b and d. Linear material models for the cornea and sclera, with *E*_cornea_ = 0.4 MPa and *E*_sclera_ = 2 MPa, were adopted for simplicity and because of the low level of pressure applied. The simulations were carried out on the finite element package Abaqus (Simulia, RI).

## 3 Results

### 3.1 Clinical and morphometric observations

Table 1 shows the results of vision testing and globe diameter measurement in 1 and 3 month old *rge* and control chickens. Data from previously characterized 9 month old birds (Boote et al., 2008) is also shown for comparison. Some minimal globe-enlargement was detected in 1 month old *rge* birds but, in contrast to previous findings (Montiani-Ferreira et al., 2003), this was not statistically significant (*p* = 0.3, two-tailed *t*-test), despite these birds being already functionally blind. In addition, physical examination of the enucleated globes did not reveal any obvious corneal flattening at 1 month. It is possible that these observations reflect differences in sampling time and tissue preservation between our study and that of Montiani–Ferreira and co-workers (using birds of the same line). Examination of 3 month old birds disclosed a 15% linear extension of the globe along the medial-lateral axis in blind *rge* birds compared to normally sighted controls (*p* = 0.00005). Moreover corneal radius of curvature appeared significantly increased, such that in *rge* eyes the corneal contour was from visual inspection essentially continuous with that of the sclera.

### 3.2 Collagen fibril organisation

**Fig. 4:**
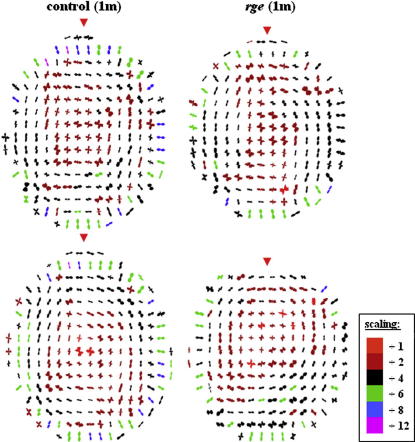
Polar plot maps showing preferred orientation of collagen fibrils in the cornea of 2 normal and 2 *rge* chickens at 1 month post-hatch. Data sampling interval is 0.4 mm × 0.4 mm. It was necessary to scale down the larger plots for montage display, as indicated in the colour key. Red arrow heads: superior globe position.

Fig. 4 shows maps of preferential collagen fibril alignment in 2 *rge* chicken corneas at 1 month post-hatch, along with 2 age-matched control corneas. In control and *rge* corneas the central-most 1.6–2 mm of tissue is characterized by a predominantly orthogonal fibril orientation, directed along the inferior-superior and nasal-temporal corneal meridians, as indicated by the approximate fourfold symmetry in the individual polar plots. Distally, the dominant fibril orientation changes in favour of circumferential/annular collagen. In the outermost cornea, regions of radial fibrillar collagen, typically more highly aligned than in more central regions (see colour key), are clearly discernable. These three discrete fibril orientation patterns are in accordance with data from fully grown normal chickens (Boote et al., 2008). However, the peripheral collagen annulus in 1 month old birds appears less highly reinforced, as indicated by the colour of the plots. Reference to Fig. 4 shows that at 1 month no significant alteration to the preferred orientation of fibrillar collagen could be detected in the *rge* cornea. A similar result was obtained from a third 1 month *rge* eye and age-matched control (data not shown).

**Fig. 5:**
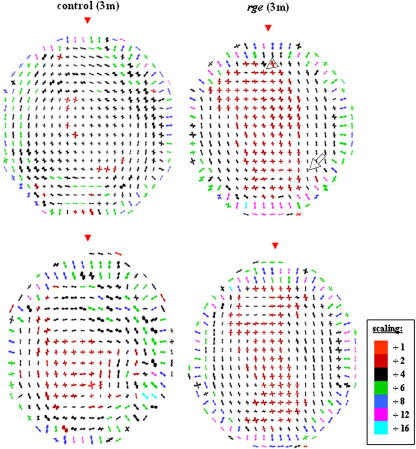
Preferred orientation of collagen fibrils in the cornea of 2 normal and 2 *rge* chickens at 3 months post-hatch, sampled at 0.4 mm intervals. The larger peripheral plots were scaled down for montage display, as indicated in the colour key. Note the disturbance to the annulus of collagen circumscribing the cornea in the *rge* birds, where the normal circumferential fibril alignment is replaced by vertical (open arrow) or orthogonal (open arrow-head). Red arrow heads: superior globe position.

**Fig. 6:**
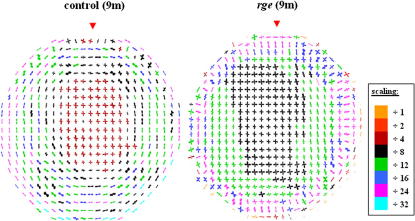
Alteration to collagen fibril orientation in *rge* continues after 3 months. At 9 months circumferential collagen in the corneal periphery is almost entirely replaced by orthogonally aligned fibrils. Data reproduced from Boote et al. (2008).

Collagen orientation at 3 months is displayed in Fig. 5. As for 1 month old birds, 3 month controls are characterized by a preponderance of orthogonal collagen in the central region, circumferential fibrils more peripherally, and radial collagen in the outermost cornea. We observed these features in a further 3 controls (data not shown). The reinforcement of annular collagen at 3 months, as indicated by the colour of the polar plots, appeared greater than at 1 month (Fig. 4) but lower than at 9 months (Fig. 6), possibly reflecting a developmental trend in the normal chick. *Rge* chicken corneas at 3 months post-hatch demonstrate similar fibril orientation patterns to controls in the central (orthogonal) and very outermost (radial) cornea. However, significant disruption to the fibril annulus is evident in the peripheral cornea, with many polar plots indicating instead an approximately superior-inferior (inter-cardinal regions—see open arrow) or orthogonal (superior/inferior regions—see open arrow-head) preferred orientation. We identified this arrangement in 3 *rge* corneas, of which data from 2 are shown (Fig. 5). For comparison, previously documented results at 9 months are reproduced in Fig. 6.

**Fig. 7:**
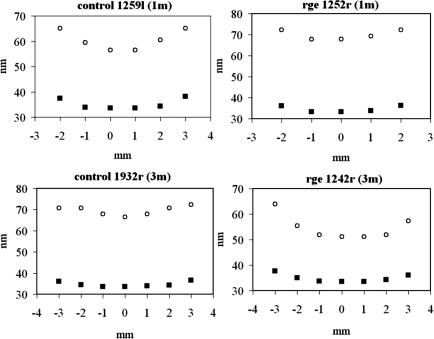
Vertical axis: collagen fibril spacing (open circles) and diameter (filled squares) along the medial-lateral meridian of *rge* and control corneas at 1 and 3 months post-hatch. Horizontal axis: distance from the corneal centre.

**Fig. 8:**
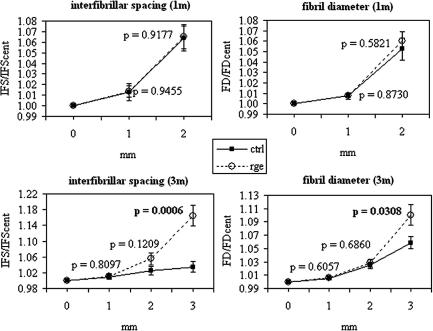
Collagen fibril spacing and diameter, as a function of distance from corneal centre at 1 and 3 months post-hatch, normalized against the central corneal value. Note the significant increase in normalized IFS and FD that occurs 3 mm from the corneal centre in 3 month old *rge* (open circles), compared to controls (filled squares).

In order to further investigate the reorganisation of peripheral collagen at 3 months, we initiated SAXS experiments to measure stromal fibril spacing and diameter in normal chickens and *rge* mutants. Collagen interfibrillar spacing (IFS) and diameter (FD) were measured at 1 mm intervals along the medial-lateral corneal meridian for 6 control and 6 *rge* eyes at 1 and 3 months post-hatch. As shown by the data sample in Fig. 7, both parameters increased distally with respect to the corneal centre. A similar observation has been documented previously for mammalian cornea (Borcherding et al., 1975; Boote et al., 2003a, 2003b), and is presumably designed to achieve an optically and/or mechanically smooth transition as the corneal collagen intermingles with the larger, more widely spaced fibrils of the sclera (Borcherding et al., 1975). For comparison of normal and mutant tissue, medial-side IFS values were averaged with corresponding values on the lateral-side for each cornea. These values were then divided by the central corneal IFS value in order to normalize against inter-specimen hydration variations (Meek et al, 1991). Results are shown in Fig. 8, alongside FD data treated similarly for convenient display. No significant difference between *rge* and controls was observed in normalized IFS or FD as a function of position at 1 month post-hatch. However, statistically significant differences were observed in 3 month samples: normalized IFS (*p* = 0.0006) and FD (*p* = 0.03) both measured higher 3 mm from the corneal centre in *rge* compared to controls. Moreover, the increase in normalized FD in *rge* appears to reflect a genuine increase in the absolute value of FD at 3 mm, since absolute FD in the central cornea appeared unchanged at 3 months (*rge*: 34.1 nm [SEM = 0.30], control: 34.3 nm [SEM = 0.26]). Furthermore, our WAXS measurements suggest that larger fibrils in the peripheral *rge* cornea do not derive from any increase in the average spacing of constituent collagen molecules (Table 2), and may point to a greater number of molecules per fibril.

### 3.3 Proteoglycan morphology and distribution

**Fig. 9:**
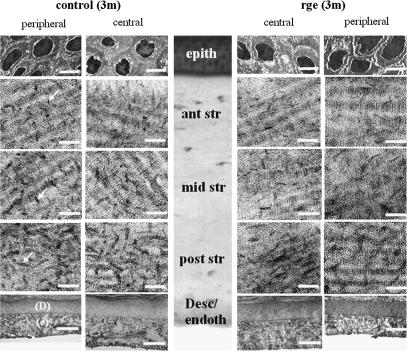
Transmission electron microscopy of central/peripheral cornea in *rge* and control birds at 3 months post-hatch. Proteoglycans (arrows) appear as dark filaments that become longer and thicker in the deepest stromal layers. Epithelial cells and endothelium (e)/Descemet’s membrane (D) are also shown. Approximate corneal depth is indicated in the central panel by histology (haematoxylin and eosine stain). Scale bars: 0.1 μm (stroma), 2.5 μm (endoth/Desc), 5 μm (epith).

TEM was employed to determine whether observed changes in IFS and FD in the *rge* corneal stroma at 3 months associate with any alteration in the general appearance of proteoglycans (PGs) in the interfibrillar space. Fig. 9 shows images of cuprolinic blue-stained sections from the central and peripheral cornea of control and *rge* chickens. Epithelium, endothelium and Descemet’s membrane appear normal in central and peripheral *rge* cornea. Throughout the stroma, PGs are evident as dark filaments associated with banded collagen fibrils (shown in longitudinal section). In both the central and peripheral control cornea PG filaments appear longer and thicker in the posterior-most 1/3 of stroma, indicating that PGs in these deeper layers may be more highly sulphated. A similar trend is evident in *rge*. No alterations in the morphology or distribution of PGs were observed in *rge* by this method.

### 3.4 Numerical simulation of tissue stress

**Fig. 10:**
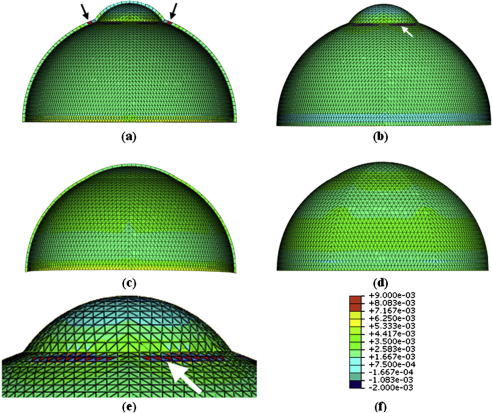
Stress distribution on the inside (a,c) and outside (b,d) surfaces of numerical simulations representing wild-type (a,b) and *rge* (c,d) chicken eyes. Limbal stress concentration (arrows) in (b) is shown expanded in (e). Stresses range between +0.009 and −0.002 MPa (f).

Fig. 10 shows the Von Mises stresses acting on the inside and outside surfaces of the numerical models representing 9 month old wild-type and *rge* chicken eyes. A concentration of elevated stress around the limbus is evident in the wild-type model (Fig. 10a,b,e—arrows), where the stresses reached 0.009 MPa compared with the 0.0008–0.0035 range observed over the rest of the anterior eye. In contrast, for the *rge* model, featuring reduced variation in curvature between the cornea and sclera, the whole anterior eye displays comparatively little variation in stress within a range of 0.002–0.004 MPa.

## 4 Discussion

**Fig. 11:**
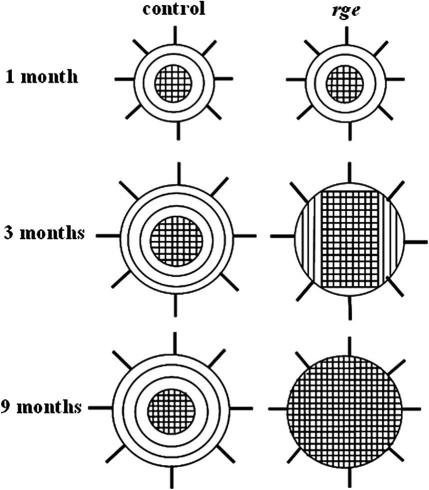
Theoretical model based on WAXS data shown in Figs. 4–6, showing fibril realignment in the post-hatch *rge* cornea. The peripheral annulus of collagen subscribing the normal chicken eye is progressively replaced by orthogonally arranged fibrils in *rge* chicks older than 1 month.

The *rge* chicken is a potentially valuable animal model for investigating how stromal ultrastructure relates to corneal shape. The results presented herein confirm alterations to peripheral collagen fibril orientation post-hatch. Furthermore, the orientation changes observed in the current study were progressive, commencing sometime between 1 and 3 months, and were concomitant with globe-enlargement and corneal flattening in homozygous birds. A gradual loss of the collagen annulus circumscribing the central cornea of the normal chicken eye was observed, in favour of collagen aligned along the superior-inferior and medial-lateral orthogonal directions. This is shown schematically in Fig. 11. Concurrent changes were also manifest in the average separation and diameter of collagen fibrils in peripheral corneal regions (Fig. 8). It is possible that these observations reflect a redistribution of peripheral lamellae, as suggested by previous data (Boote et al., 2008), particularly as the current study did not identify any alteration to distal collagen organisation at the molecular level (Table 2), and central fibril diameter was unaffected. Moreover, the changes in size and spacing of peripheral fibrils did not appear to be due to any gross alteration to stromal PGs, although it is important to note that no selective staining of PG sub-types was performed here.

It is likely that significant collagen ultrastructural changes will affect corneal biomechanics. Indeed, it has been shown (Boote et al., 2005) that the principal factors which determine regional corneal elastic modulus are: (1) collagen fibril orientation with respect to the measurement direction, (2) fibril volume fraction, and (3) fibril elastic modulus. We have here demonstrated changes to preferential fibril orientation and fibril size/spacing in the peripheral *rge* cornea, that may be expected to alter the first and second of these factors respectively, and hence regional tissue mechanics. In the normal cornea the presence of an annular fibril structure at the limbus, the junction between the cornea and the surrounding sclera, is thought to be of particular mechanical importance (Maurice, 1984; Radner et al., 1998; Aghamohammadzadeh et al., 2004; Boote et al., 2004). The absence of circumferential fibrils in the *rge* eye after 1 month post-hatch was the most striking result of the present study. We propose that the loss of this structure might relate to the corneal flattening observed in *rge* mutants.

In order to test this hypothesis, we simulated numerically the predicted stress distribution in the cornea and anterior sclera of 9 month old wild-type and *rge* chicken eyes (Fig. 10), using simplified geometrical constructs of the anterior eyeball (Fig. 3). The simulations show a clear stress concentration in the limbal region of the normally shaped wild-type eye (Fig. 10a,b,e—arrows). This is consistent with the theory of Maurice (1984), who suggested that the fusion of the cornea and sclera must necessarily result in locally increased circumferential tension purely on account of their significantly different curvatures. Notably this limbal stress concentration is absent in the misshapen *rge* eye at the same age (Fig. 10c and d). In light of the different fibrillar arrangement of the wild-type and *rge* eye at 9 months (Figs. 6 and 11), two alternative scenarios could be envisaged that are consistent with this observation from biomechanical considerations. The first possibility is that the loss of reinforcing circumferential fibrils, and consequent failure of the eye to carry the increased limbal tension, might force the cornea to flatten in order to reduce the stress at the limbus. Alternatively, if the *rge* eye adopted (for some other reason) a shape in which there was little variation in curvature between cornea and sclera, the resulting reduced limbal stress might then encourage the eye to reduce the density of circumferential fibrils there as their reinforcement is no longer required.

It remains to be established exactly how corneal changes in *rge* relate to the globe enlargement and vision loss (via retinal degeneration) presented by the disease. From the current data, globe and corneal changes occur concurrently after 1 month post-hatch, suggesting that these events may be related and are likely triggered by the loss of functional vision which occurs around this time (Montiani-Ferreira et al., 2003). Moreover, corneal collagen reorganisation appears to be restricted to the periphery of the tissue, which might suggest that corneal changes are secondary to events in the sclera. In myopia progression, globe elongation is accompanied by manifold changes in the scleral extracellular matrix (Liu et al., 1986; Funata and Tokoro, 1990; Norton and Rada, 1995; Rada et al., 2000; McBrien et al., 2001; McBrien and Gentle, 2003). It is possible that morphometric changes in blind *rge* birds are triggered by retinal signals via mechanisms similar to those observed in chick form-deprivation myopia/hyperopia (Gottlieb et al., 1987; Irving et al., 1992; Troilo et al., 1995), where globe enlargement and corneal reshaping are also induced by deprivation/defocus of the retinal image. Furthermore, although there exists no ultrastructural information on myopic cornea, there is some documented evidence for corneal biomechanical change in humans with high myopia (Shen et al., 2008).

Some of the morphometric changes in the *rge* eye are akin to those seen in chicks subjected to constant light exposure (Li et al., 1995), and could suggest a similar hormone-driven mechanism of abnormal ocular growth for *rge*. Chick eye growth is regulated by the antagonistic effects of dopamine and melatonin (Schaeffel et al., 1995). Reduced retinal melatonin levels have been reported in the “constant light chick” (Zawilska and Wawrocka, 1993; Li et al., 2000) and are associated with elevated scleral growth, but whether hormone levels are affected in *rge* awaits further investigation.

Characterization of corneal matrix metalloproteinase and/or cellular fibroblast activity may provide future insight into whether any active matrix remodelling is occurring in the *rge* cornea, while the origin of increased central corneal thickness in *rge* birds remains similarly unexplained. In addition, a correlative study of corneal topography with ultrastructure, along with direct measurement of corneal material properties, would be a minimum requirement in order to establish definitively whether a dependency between corneal structure and topography exists in *rge*. Moreover, results of IOP measurements in *rge* are conflicting (Inglehearn et al., 2003; Montiani-Ferreira et al., 2003), and a potential IOP-mediated effect on corneal curvature still presently cannot be ruled out. However, from the standpoint of tissue biomechanics, the data presented in this paper support an association between corneal collagen reorganisation and shape changes in the *rge* chicken eye, enhancing its potential as a useful animal model for investigating ocular function.

## Figures and Tables

**Table 1:** Details of 1- and 3-month-old control/*rge* chicken eyes used in the current study, and comparison with previously studied 9 month old samples. WL, white Leghorn; IB, Isa brown.

Type (breed)	Age (months)	Vision	Medial-lateral globe diameter, mm (SEM)	*n*
Control (WL)	1	Sighted	14.0 (0.2)	12
*rge* (WL)	1	Blind	14.6 (0.6)	6
Control (WL)	3	Sighted	18.0 (0.3)	11
*rge* (WL)	3	Blind	20.7 (0.3)	6
Control (IB/WL)⁎	9	Sighted	18.0 (0.0)	3
*rge* (WL)⁎	9	Blind	23.0 (1.0)	3

⁎ Data reproduced from Boote et al. (2008).

**Table 2:** Intermolecular Bragg spacing as a function of radial position along the medial-lateral meridian of control and *rge* corneas at 1 and 3 months post-hatch.

Age	mm	Intermolecular Bragg spacing, nm (SEM)		*n*	*p*
Control	*rge*
1 m	0	1.55 (0.004)	1.55 (0.005)	3	0.899
1	1.54 (0.009)	1.54 (0.008)	6	0.875
2	1.54 (0.008)	1.54 (0.006)	6	0.724

3 m	0	1.55 (0.000)	1.54 (0.005)	3	0.824
1	1.54 (0.003)	1.54 (0.003)	6	0.674
2	1.54 (0.004)	1.54 (0.005)	6	0.204
3	1.54 (0.005)	1.53 (0.000)	6	0.559
